# Supplementary material for: Are carbon emissions trading and green financial instruments synergistic? -Comprehensive quantitative research based on content analysis
Source: PLoS One. 2024 Mar 7;19(3):e0298601. doi: 10.1371/journal.pone.0298601 (PMC10919601; doi:10.1371/journal.pone.0298601)
Supplement: S1 Data — (ZIP) [file pone.0298601.s001.zip › Data/地级市Z_F工作报告.zip!/╔╜╢1⁄2/╠⌐░▓╩╨/└┤╫╘╣1⁄2╓┌║┼í╛┬φ┐╦╩2╛▌═°í┐_2017.docx]

各位代表：

现在，我代表市人民政府向大会报告工作，请予审议，并请市政协委员和列席会议的同志提出意见。

十六届政府工作回顾

十六届政府以来，在中共泰安市委的坚强领导下，在历届班子打下的良好基础上，全市上下攻坚克难，开拓创新，真抓实干，圆满完成了“十二五”规划，实现了“十三五”规划良好开局，经济社会发展取得显著成绩。

（一）经济平稳较快发展，综合实力明显提高。全市生产总值跨过3000亿元大关，达到3316.8亿元，是2011年的1.5倍，年均增长9.2%。一般公共预算收入突破200亿元，达到206.7亿元，年均增长8.4%。全社会用电量、工业用电量年均增长4.95%和3.78%。实现社会消费品零售总额1462.8亿元，年均增长12.6%。金融机构各项存贷款余额分别达到3056.2亿元、1936.7亿元，均比2011年翻了一番。

（二）经济结构不断优化，转型升级明显加快。三次产业结构由2011年的9.3：52.2：38.5调整为8.5：44.8：46.7，实现了由“二三一”向“三二一”的历史性转变。工业经济提质增效。规模以上工业主营业务收入、利税分别达到5885.4亿元、543.4亿元，是2011年的1.2倍和0.95倍。着力构建“十大”产业体系，全市装备制造业增加值占规模以上工业增加值的比重达到39.8%。累计完成技改投入3498.7亿元，完成技改项目1602项。新增中国驰名商标26件、山东名牌产品26个。鲁能泰山电缆、航天特车两家企业获省长质量奖提名奖。我市入选国家首批新能源示范城市，泰安高新区被确定为国家首批分布式光伏发电应用示范区，新泰市被列为全国首批采煤沉陷区光伏领跑技术基地。

现代农业加快发展。有机蔬菜、奶牛存栏、名优果品、苗木花卉、茶叶种植分别达到36万亩、20.3万头、170万亩、41.3万亩和3.8万亩。新发展规模以上农业龙头企业、农民合作社、家庭农场292家、4688家和3155家。泰安农高区升级为国家农业科技园区，岱岳区被列为国家级现代农业示范区，肥城市被确定为国家级出口食品农产品质量安全示范区。投资135亿元完成国家小农水重点县建设、水库除险加固、河道治理、山洪灾害防治、农村饮水安全等各类水利工程35130项。累计新改建农村公路3339.3公里，提前4年完成农村公路安全生命防护工程。农作物耕种收综合机械化水平达到86.6%，比2011年提高6.2个百分点。

服务业发展势头强劲。全市实现服务业增加值1550.4亿元，是2011年的1.8倍，连续6年被表彰为全省服务业发展先进市。年接待游客连跨3个千万人次台阶，突破6000万人次，旅游消费总额突破600亿元，年均分别增长11%和15.3%。太阳部落、宝泰隆旅游度假区、刘老根大舞台、大汶口国家考古遗址公园、泰山温泉城等建成开业；蓝海大饭店、宝盛大酒店、万达嘉华酒店等投入运营；天颐湖旅游度假区、莲花山旅游度假区、白佛山旅游区等一批旅游大项目加快推进。我市入选国家全域旅游示范区创建名单，旅游业正逐渐由单日游向多日游转变。全市电子商务交易额达到727亿元。新发展现代物流园区20个、规模以上物流企业192家；我市被确定为全国冷链物流发展示范城市，新矿物流中心进入全国物流企业百强。金融商务区加快建设。泰山国际会展中心建成运营。信息服务、健康养老、家政服务等新兴服务业快速发展。

创新驱动战略深入实施。全市实现高新技术产业产值1727.5亿元，占规模以上工业总产值的比重比2011年提高7个百分点。我市3个项目入选全国首批46个智能制造试点。康平纳集团获国家科技进步一等奖，岱银集团获中国专利金奖。国家级高新技术企业发展到119家，比2011年增加60家。国家级创新研发平台达到8家。国家再制造机械产品质检中心建成。“泰山创客空间”“钛创空间”等6家众创空间通过科技部备案。泰安高新区升级为国家级高新技术产业开发区。

（三）重点项目加快建设，发展后劲明显增强。全市固定资产投资累计完成11576.56亿元，年均增长15.4%。共实施市级重点建设项目618个，完成投资2381.3亿元，其中亿元以上项目604个。泰安如意科技时尚产业园、中材玻纤新材料工业园、北汽国泰汽车产业园、瑞福锂业碳酸锂项目、海斯摩尔医卫制新材料产业园、瑞星粉煤气化工程等加快建设。盛德大业金属新材料、3D打印增材制造、晶泰星光电科技、威高集团高端医疗器械等新兴产业项目落地建设。

（四）改革开放持续深化，发展活力明显提升。制定实施供给侧结构性改革50条意见，各项改革任务顺利完成。深化“放管服”改革，连续5轮取消、下放行政审批事项96项。深入推进商事制度改革，新登记各类市场主体13.22万户。探索建立“三位一体”国资国企改革架构，实施市属国有企业负责人经营业绩考核和薪酬管理制度，累计上缴国有资产收益10.54亿元。国库集中支付、政府采购、投资评审等改革深入推进。“营改增”试点全面推开，连同其它税收优惠政策为企业减税53.7亿元。政府与社会资本合作取得积极成效，宁阳县经验做法在全国推广。党政机关公务用车制度改革顺利完成。不动产统一登记全面实施。6家县级农村信用合作社全部改制为农村商业银行。新型农村合作金融试点实现县域全覆盖。农村土地承包经营权确权登记颁证工作荣获省优秀等次。肥城市被确定为全国农民住房财产权抵押贷款试点县。农村集体产权制度改革、集体林权配套改革深入推进，新泰市被列为全国集体林业综合改革试验示范区。国有林场改革试点走在全国前列。供销社改革走在全省前列。文化体制、社会治理、社会事业等其它领域改革不断深入。

对外开放进一步扩大，五年累计批准外资项目300个，实际利用外资19.7亿美元，年均增长48.8%；完成出口总额77.9亿美元，年均增长7.1%；完成对外承包工程营业额12.33亿美元、境外投资6.42亿美元。泰安高新区海关综合监管区、青岛保税港区泰安（新泰）功能区加快建设。我市被评为全国外贸竞争力百强城市。

（五）基础设施加快完善，城乡面貌明显变化。城乡规划体系更加完善。全市常住人口城镇化率、户籍人口城镇化率达到59.06%、50.25%，分别比2011年提高7.64和15.65个百分点。泰城建成区面积达到130.6平方公里，中心城区人口突破100万。我市被省政府确定为大城市试点。大力实施片区开发、组团建设和功能提升，泰城中心城区改造力度加大。高铁新区开发加速推进，展现现代气息，呈现崭新面貌。徂汶景区加快建设，促进了泰城由“依山而建”向“依山傍水”发展。中央商务区、新华片区等城市片区和万达广场、天房美郡、鲁商国际、五矿万境水岸等城市综合体顺利建设。104国道改线、泮河大街改造、灵山大街、万官大街等一批道路工程顺利实施。引水进城、供热“汽改水”、供电“多表合一”、三合水厂改造、污水处理厂升级等一批工程项目建成启用。调整优化泰城城市管理体制，下大气力解决了一大批长期影响市容市貌的突出问题，成功通过国家卫生城市复审。全面加强生态泰安建设，空气质量居全省前列，主要河湖水质保持稳定，森林覆盖率达到39.6%，城乡环卫一体化实现全覆盖。首创3处国家湿地公园，新增1处国家森林公园，泰山、徂徕山入选全国首批“中国森林氧吧”，泰山区被确定为国家重点生态功能区。我市被授予中国人居环境奖、国家森林城市、国家节水型城市等荣誉称号。

（六）社会事业蓬勃发展，社会治理明显加强。投入38.3亿元新建、改扩建学校273所，城镇中小学“大班额”问题得到初步缓解。投入13.2亿元为382所学校购置教学设施设备，农村义务教育薄弱学校办学条件明显改善。六个县市区全部通过国家义务教育均衡发展验收。泰安一中新城校区和市特教中心基本建成，即将投入使用。基层医疗卫生机构综合改革全面完成，14家县级公立医院实施了综合改革试点，基本药物制度实现全覆盖，基本公共卫生服务人均项目经费由2011年的25元提高到45元。启动实施19家城市公立医院综合改革，全面取消药品加成，分级诊疗等各项政策得到落实。市妇幼保健院新院建成启用。市中心医院高新区院区加快建设。两孩政策全面实施。泰安市文化艺术中心投入使用。建成乡镇综合文化站86个、基层综合性文化服务中心2567个。泰山区、新泰市、肥城市入选全国文化先进县。东平戴村坝列入世界文化遗产，泰山皮影列入联合国非物质文化遗产。我市被表彰为全国文明城市提名城市，第六次荣获全国双拥模范城称号。体育事业取得新成绩。对口援建新疆岳普湖县和重庆巫溪县工作成效明显。国防动员、人民防空、民兵预备役工作顺利开展。妇女儿童、老龄工作有新提高。外事侨务、民族宗教、气象、水文、防震、史志、档案等社会事业全面进步。

“六五”普法圆满完成，“七五”普法、依法治市工作顺利启动。我市被确定为首批全国社会组织建设创新示范区，菜单式志愿服务模式、城市社区治理创新、农村社区服务体系建设经验在全国推广。平安泰安建设深入推进，“天网工程”、大数据警务云体系、民生警务平台有效运行，消防工作在全省保持先进。食品药品市县乡村四级监管体系初步形成，监管能力不断提升。探索建设“安如泰山”科学预防体系，我市被列为全国遏制重特大事故试点城市，安全生产工作走在全国前列。

（七）民生得到有效保障，群众生活明显改善。全市累计各项民生支出1129.6亿元，占一般公共预算财政支出的78.2%。城镇和农村居民人均可支配收入分别达到30299元、14428元，年均增长9.5%和11.3%。扎实推进精准扶贫、精准脱贫，全市30.56万贫困人口实现脱贫，库区移民避险解困工程顺利实施。五年新增城镇就业34.08万人、农村劳动力转移就业44.81万人，被国务院表彰为全国创业先进城市。累计收缴各项社会保险费649.66亿元，发放各项社会保险待遇548.96亿元。社会养老保险制度实现全覆盖，居民养老保险基础养老金标准由每人每月55元提高到100元。城乡居民医疗保险制度实现并轨。城乡低保、农村五保供养标准大幅提高。市残疾人康复中心完成建设。建成城市社区老年人日间照料中心193处、农村幸福院1063处，养老床位达到3.5万张。累计发放住房公积金贷款74亿元。建设各类保障性安居住房10.6万套（户）。完成农村危房改造19687户。泰城新增公交1286辆，实施了“一小时免费·优惠换乘”、延时服务等惠民举措。

（八）政府自身建设不断加强，发展环境明显优化。扎实开展党的群众路线教育实践活动、“三严三实”专题教育和“两学一做”学习教育，严格落实中央八项规定精神，政风行风持续好转。建立政府法律顾问制度，法治政府建设取得新成效。加强行政审批事中事后监管，编制公布行政审批事项目录、行政权力清单、政府部门责任清单。探索实施建设项目“一窗受理”代办服务机制和“容缺预审”绿色通道制度。建立市级公共资源交易平台，完成市县两级政务服务平台建设。认真开展领导干部经济责任审计、纠风专项治理、政风行风评议，强化“一岗双责”，政府廉政建设得到加强。持续抓好政务督查、应急管理、12345民生服务平台、工作日志等工作，行政能力和服务水平不断提升。

各位代表，五年的发展有目共睹、来之不易。这是市委坚强领导，历届政府接续努力的结果，是市人大、市政协及社会各界有效监督、鼎力支持的结果，是全市人民锐意进取、拼搏实干的结果。在此，我代表市人民政府，向全市广大干部群众、各民主党派、工商联、无党派爱国人士，向人民解放军和武警驻泰部队及驻泰单位，向关心支持泰安发展的港澳台同胞、海外侨胞及海内外朋友，表示崇高的敬意和衷心的感谢！

各位代表，五年的发展成绩斐然，但存在的矛盾和问题仍然不少。主要是：经济总量偏小，综合实力还不强；经济运行不稳定，实体经济发展动力不足；工业仍是经济发展的最大“软肋”，产业层次低、实力弱、竞争力不强；科技创新能力不足，高新技术产业规模小、占比低，高层次创新平台少；财税收入总量小、质量不够高，财源建设需要发力；地区发展不平衡，中心城区强县市弱，群众对经济发展还有很多“期待”。对外开放水平较低，改革力度不够大，发展中还存在不少体制机制障碍，群众对改革开放还有很多“期望”。城市建设管理还不够到位，生态环境治理力度还需加大，文化、教育、医疗、交通、住房等民生保障还有一些欠缺，群众对此还有很多“期盼”。公共服务水平还有短板，发展环境不够优化，政府服务效能还不高，群众对此还有一些“期许”，等等。对于群众的这些期待、期望、期盼和期许，我们必须高度重视，采取有力措施，尽心尽力回应好、解决好。

新一届政府工作总体要求和目标

新一届政府五年,是全面建成小康社会的决胜阶段。市十一次党代会科学研判、审时度势，提出了“践行新理念、增创新优势、建设新泰安”的主题，这是未来五年全市上下思想和行动的根本指导，也是政府工作的基本遵循。新一届政府必须以坚定的政治定力、强烈的责任意识，抢抓机遇，乘势而上，不断开创新局面。

今后五年政府工作总体要求是：高举中国特色社会主义伟大旗帜，以邓小平理论、“三个代表”重要思想、科学发展观为指导，深入贯彻习近平总书记系列重要讲话精神，按照中央和省市委决策部署，围绕统筹推进“五位一体”总体布局和协调推进“四个全面”战略布局，牢固树立创新、协调、绿色、开放、共享发展理念，坚持以人民为中心的发展思想，把握稳中求进工作总基调，适应引领经济发展新常态，牢牢抓住发展第一要务，以供给侧结构性改革为主线，着力强化创新驱动，着力深化改革开放，着力加强生态环保，着力促进协调共享，践行新理念，增创新优势，努力建设富裕文明幸福新泰安。

今后五年政府工作总体目标是：圆满完成“十三五”规划目标任务，提前全面建成小康社会，加快建设富裕文明幸福新泰安，为迈向基本实现现代化新征程奠定坚实基础。这是经济社会发展进入新阶段的内在要求，是更高水平、更高层次的发展目标，是中国梦的泰安篇章，主要体现在以下几个方面：

——更有质量、更高效益的发展新格局。全市经济保持中高速增长，主要经济指标增速高于全省平均水平。城乡居民收入增幅高于地区生产总值增幅，农村居民收入增幅高于城镇居民收入增幅，税收占财政收入、主体税收占税收收入的比重显著提高。产业迈向中高端水平，供给侧结构性改革成效明显，转型发展取得重大进展，开放型经济迈上新台阶。提前实现地区生产总值和城乡居民人均收入比2010年翻一番。

——更有活力、更加向上的文明新气象。中国梦和社会主义核心价值观不断深入人心，以泰山文化为核心的优秀传统文化进一步弘扬，人民群众思想道德、科学文化、健康素质和全社会文明程度显著提高。文化事业繁荣发展，文化产业不断壮大。诚信体系更加完善。人民民主更加健全。社会治理现代化水平全面提升，社会大局和谐稳定。全市上下人心思上、风正气顺、心齐劲足、充满活力。

——更加宜居、更可持续的美丽新家园。新型城镇化和城市现代化水平明显提高，空间布局持续优化，基础设施配套完善。生态泰安建设扎实推进，资源节约、环境保护和污染治理取得更大进展，大气、水和土壤环境质量显著改善，森林覆盖率继续提升。生产方式、生活方式和消费模式向绿色转变。泰安的天更蓝、水更绿、山更青、路更畅，城乡环境更优美。

——更多实惠、更高品质的幸福新生活。脱贫攻坚任务如期完成，现行标准下的贫困人口全部脱贫，经济薄弱村全部脱困。全覆盖、多层次、均等化公共服务体系基本形成，文化、教育、就业、医疗、住房、社会保障、社会救助等各项事业都有新发展。群众生活更有质量、更有尊严，获得感、幸福感和满意度不断提升。

各位代表，实干推动崛起，奋斗创造辉煌。我们相信，只要全市上下始终践行五大发展新理念，不断增创厚植发展新优势，坚定信心，振奋精神，苦干实干，就一定能把宏伟蓝图变成美好现实！

2017年政府工作安排

2017年是新一届政府的起始之年，也是为“十三五”发展夯实基础的关键一年，开局谋势，气势要足，发力要准。按照市委“四个一”总体安排部署，今年全市经济社会发展主要预期目标是：生产总值增长7.5%左右；一般公共预算收入增长6.5%左右；固定资产投资增长11%左右；社会消费品零售总额增长10.5%左右；外贸进出口保持稳定；城镇居民人均可支配收入增长8%左右，农村居民人均可支配收入增长8.5%左右；服务业增加值占比提高1.5个百分点左右；研发投入占GDP比重高于全省平均水平；常住人口城镇化率和户籍人口城镇化率均提高2个百分点；全面完成省政府下达的节能减排降碳等约束性指标。实现上述经济社会发展目标，必须要有强大的动能和引擎，必须聚焦用力增创“五大发展新优势”。

（一）增创工业经济发展新优势，充分发挥其主体支撑作用。工业化是经济社会发展不可逾越的重要阶段，没有工业化就没有城市化。工业实力仍然是一个地区竞争力的重要标志，尤其对于我们泰安工业化水平偏低的地区，面临着国家财税体制的重大调整，在今后相当长的时期内，工业经济仍将具有主导作用、处于主体地位，必须进一步强化工业强市理念，实施工业强市战略，推动泰安工业转型升级、做大做强。在发展布局上，重点实施好“一高、三强、两突破”。做好“一高”文章，即泰安高新区（包括泰山区和岱岳区的工业园区），重点发展高新技术产业、高端装备制造业。培植“三强”企业，即重点培植发展一批区域拉动力强、行业竞争力强、财税贡献能力强的“三强”企业，引导生产要素向“三强”企业聚集、优惠政策向“三强”企业倾斜、人才资源向“三强”企业汇聚，带动各县市区分别构建起由5家以上税收过亿元大企业拉动、10家以上税收过5000万元骨干企业支撑、30-50家成长型中小企业撬动的企业组织格局，凝聚产业转型升级的强大合力。实现“两突破”，即突破新泰、肥城工业的龙头带动作用，支持两市发挥经济总量大、产业基础雄厚、各方面人才聚集等比较优势，制定具体方案，落实相应政策，创新发展举措，提升产业水平，尽快实现突破发展。支持宁阳县、东平县结合各自实际，发挥各自特色，制定有效措施，在骨干企业发展和中小企业培育上下功夫、求突破，不断壮大工业规模实力。大力推动大众创业、万众创新，全面落实支持民营经济发展各项政策措施。加大技改投入，推进“两化融合”。继续实施规模企业规范化公司制改制，引导建立现代企业制度，夯实对接资本市场基础，年内完成改制企业150家以上。高度重视企业家队伍建设，实施企业家素质提升工程，完善与非公有制企业和商会协会联系沟通机制，在全市形成关心、爱护、支持、包保、协调、联络企业家的浓厚氛围，造就一支视野开阔、具有战略眼光，勇于进取、富有社会责任感的企业家队伍。弘扬坚守实业、精益求精的“工匠精神”，大力实施工业质量品牌发展战略，推动泰安制造向泰安智造转变、泰安产品向泰安品牌转变。

（二）增创旅游经济发展新优势，充分发挥其王牌牵引作用。旅游是幸福产业之首，也是我市经济发展的最大优势和王牌。必须牢固树立“大景区、大旅游、大项目、大目标”意识，强化省会文化旅游功能区定位，全力推进旅游业转型发展，加快建设国际著名遗产地和旅游目的地城市。重点依托“两山一河”旅游发展框架和布局，力争5年内着力开发建设投资过100亿元的南北两大景区：北部，建设以泰山东麓“泰山慢谷”为主题的大景区，在重点对泰山核心景区保护提升的基础上，加快外延景区的转型发展，不断生成新的增长点和拉动力；南部，建设以徂徕山大汶河“健身休闲”为主题的大景区，重点突出生态、旅游、总部经济等新型业态，拉开框架，打好基础，完善功能，集聚项目，形成新的增长极。

大力实施“旅游+”发展战略，努力创建国家全域旅游示范市。积极拓展旅游业态，打造一批旅游特色小镇和乡村旅游度假区，推出“泰山人家”等高端品牌。通过资本运作、招商引资等途径，培育壮大一批带动力强的旅游骨干企业。扩大“平安泰山”品牌知名度和影响力，打造中国山岳旅游第一名片。加大旅游市场整治力度，加快旅游云数据中心、智慧旅游服务平台、旅游集散中心等工程建设，实施旅游服务标准化，不断提升旅游业发展的环境和条件。

旅游业是我市服务业的龙头，与其它各类服务业发展相辅相成、互相促进，必须一体推进。着力推动服务业三年行动计划和18个重点行业转型升级方案落实，促进服务业拓宽领域、优化结构、提升层次。突出生产性服务业与制造业深度融合，着力抓好现代物流、电子商务、研发创意、信息服务等产业提档升级、加速发展。把会展经济摆在更加重要位置，重点培育具有较强影响力的特色会展品牌，尤其着力打造“旅游+会展”发展模式，推动会展业和旅游业融合发展。推动文化“走出去”，加快发展影视制作、动漫游戏等文化产业新业态，培育大型文化企业集团，推进文化产业园、创意街区建设。以建设知名体育休闲城市为目标，加快体育产业发展。积极推进医药研发、健康养生、康复护理等大健康产业和养老服务业发展，建设医养融合产业发展新高地。

（三）增创城市经济发展新优势，充分发挥其龙头带动作用。我市生态条件好，文化旅游资源丰富，县大镇大村大、人口聚集度高，与省会同城化因素多，加快推进城市化进程优势明显。以实施全省大城市试点为契机，强化规划引领，优化城市布局，完善城市功能，提升城市形象，努力打造山水名城新品牌。按照区域性中心城市发展定位，推进泰城新一轮城市总体规划修编，进一步拉开发展大框架。近两年，泰城重点开发建设两大区域：一是依托东部济泰高速公路，隆起拓展东部新城，即借助济泰高速公路进入泰城后的五个立交出口等关键节点，发挥我市资源和环境优势，面向省会，立足高校等创新创业人才，科学布局产城融合、宜业宜居总面积约30平方公里的生态化、节点式新型社区和新型园区，建成集旅游休闲、科技研发、高端制造、生态绿地为一体的现代化产业新城。二是依托西部京沪高铁，改造提升西部新城，即在完善西部高铁新区25平方公里开发建设的基础上，升级改造25平方公里旅游经济开发区，南北融合形成50平方公里的现代化宜居新城。按照产城融合发展思路，支持岱岳区抓好京沪高铁以西、新104国道以东约100平方公里的“一城一区”规划建设，培育区域核心增长极。同时，加快泰肥一体化发展，在规划制定、产业互补、交通基建、公共服务、要素配置等方面，向同城化大步迈进。其它县市区也要以新城区、工业园区和交通干线沿线为重点，进一步拉开城市框架，拓展城市空间。

加大泰城中心城区投入力度，实施老城改造和古城修复，完善城市服务功能。加快推进财源街中央商务区、历史文化轴、时代发展线、城市主要内外环线、主要出入口改造提升，以及城市功能区之间互联互通主干道路等重点工程。加大城中村、老旧小区、棚户区改造力度，年内实施棚改3.4万套。抓好城市供热、供气等技术改造，扩大集中供热、供气覆盖面。加强城市雨污分流管网改造和排水防涝设施建设。加快建设海绵城市和地下综合管廊等重大设施。建设智慧城市，构建智能交通、智能管网、智能城管、智能安全保障体系。开展城市违法建设治理行动。加大黄前水库水源地整治保护力度。大力实施“绿亮净美”工程，加快全国文明城市创建步伐。

扎实推进“三个市民化”进程，全面实施流动人口居住登记和居住证制度，年内确保13.8万农业转移人口实现市民化。实施中小城市和特色小镇培育计划，加快形成一批“产业特色突出、人口聚集度高、设施配套齐全、生态环境优美”小城镇，建设一批“商贸带动型、工业带动型、旅游带动型和交通带动型”特色小镇。重点抓好满庄镇、楼德镇、石横镇、磁窑镇、银山镇、西张庄镇等国家级重点镇和示范镇建设；培育建成徂汶景区汶水小镇、新泰市羊流智能起重小镇、岱岳区大汶口水上石头古镇、东平县老湖水浒影视小镇等4个省级特色小镇。积极推进农村新型社区建设，深化农村环境综合整治，巩固城乡环卫一体化成果，加强乡村风情风貌保护，打造一批美丽乡村。

（四）增创农业融合发展新优势，充分发挥其强基固本作用。深入实施藏粮于地、藏粮于技战略，加强耕地数量、质量、生态“三位一体”保护。增加绿色优质农产品供给，狠抓农产品标准化生产，大力发展有机蔬菜、泰山茶、畜禽养殖、苗木花卉、名优经济林等优质高效特色产业，力争蔬菜面积稳定在200万亩，泰山茶面积达到4.2万亩。积极培育“新六产”，加快“一产四化”进程，探索发展“互联网+现代农业”，创新发展生态休闲农业，促进农村一二三产业融合。将农业龙头企业、农民合作社、家庭农场作为融合发展三大主体，加大引进培育力度，年内新上、扩建规模以上农业龙头企业50家，新增农民合作社200家、家庭农场100家。加强农产品质量安全监管体系建设，深入开展农产品质量安全县创建活动。大力实施农业品牌化战略，加强农产品地理标志建设，培育农产品区域公共品牌和企业知名品牌。深化农村土地所有权、承包权和经营权“三权分置”，抓好土地经营权和住房财产权抵押贷款试点。全面铺开农村集体产权制度改革，抓好农村产权交易市场建设。巩固提升国有林场改革成果。深化供销社综合改革。全面推进农业水价综合改革和“河长制”。加大农业基础设施投入力度，抓好王家院水库建设、“引黄入泰”、彩山和角峪水库串联扩容改造、东平湖除险加固等重点水利工程。推广大型化、高新化新技术新机具，加快农作物全程机械化发展。

（五）增创社会和谐发展新优势，充分发挥其民生保障作用。任何时候都要把民之所望作为政之所向，让人民群众有更多的获得感和幸福感。坚持精准扶贫、精准脱贫方略，深入推进产业发展、金融扶贫、医疗救助、兜底保障等脱贫攻坚行动，建立可持续脱贫机制，稳定实现建档立卡贫困人口脱贫。坚持搬迁社区和产业园区“两区共建”，统筹推进移民避险解困、黄河滩区迁建、易地扶贫搬迁三项工程，确保搬迁群众稳定脱贫。千方百计扩大就业，重点抓好高校毕业生就业、复退军人安置、农村劳动力转移和去产能职工转岗再就业工作，引导毕业生到基层、新兴领域、小微企业就业，年内确保城镇新增就业5万人以上，城镇登记失业率控制在3.5%以内。

深入实施全民参保登记计划，着力解决断保断缴问题。积极推进养老保险制度改革，完善参保缴费政策，稳步提高基本养老金和基础养老金标准。加快完善医疗保险政策，加强医保基金监管。完善居民大病保险制度，全面实施职工大病保险。积极开展职工长期护理保险试点。推动农村低保与扶贫开发政策有效衔接，提高农村低保标准。大力发展福利慈善事业，健全农村留守妇女、儿童和老年人关爱服务体系。开展残疾人精准康复服务行动。加快建设社会养老服务体系。

深化基础教育综合改革，实行中小学校长职级制，完善学校内部治理结构。继续推进“全面改薄”工作，新增校舍8.8万平方米。新建、改扩建学校223所，基本解决城镇中小学大班额问题。完善学生资助体系，做好家庭经济困难学生资助工作。加快现代职业教育体系建设，大力支持驻泰高校改革发展，打造高层次人才聚集高地。鼓励社会力量兴办教育。全面推进城市和县级公立医院综合改革，建立健全分级诊疗制度，完善法人治理结构。发挥市公立医院药品医用耗材采购联合体作用，降低采购成本，减轻群众就医负担。稳妥实施“全面两孩”政策，深化计划生育服务管理。加强重大疾病综合防治，促进中医药事业发展。健全现代公共文化服务体系，扎实开展文化惠民活动。加快泰安市博物馆新馆和泰山文化生态保护区建设。大力发展体育事业和体育产业。深入开展民族团结进步创建活动，依法管理民族宗教事务。认真抓好国防动员、人民防空、民兵预备役、双拥共建等工作。繁荣发展其它社会事业。

深入推进平安泰安建设，完善立体化、信息化社会治安防控体系，创新社会矛盾预防和化解机制。深化司法体制改革，统筹推进公安基层基础建设，夯实社会治安管理工作根基。全面落实安全生产企业主体责任，一手抓科学预防体系建设，一手抓隐患排查整治，力争不发生较大以上事故。加大食品药品监管力度，深入开展“食安泰安”品牌建设，全面推进食品安全城市创建工作。健全应急事件预防预警和应急处置体系，提高应急管理和处置能力。

绿水青山就是金山银山，生态环境也是最大民生，必须久久为功、加大力度，为子孙后代造就宜居美丽家园。大力实施大气污染防治“蓝天工程”，健全联防联控机制，扎实做好重污染天气应对工作，形成区域治霾合力；切实抓好城市面源污染防治，加强扬尘整治和渣土运输管理；积极实施“工业绿动力”计划，加快发展新能源，全面推进燃煤电厂、锅炉超低排放，持续改善空气质量。大力实施“碧水行动”计划，集中开展城乡河道整治行动，同时以重要饮用水水源地为重点，全方位保护水环境质量，努力创建全国水生态文明城市。大力实施“绿满泰安”行动，全面提升国家森林城市建设水平。实施更加严格的生态保护制度，健全网格化环境监管体系，加大环境保护执法力度。

增创新优势、培植新动能，必须牢牢把握好改革开放、科技创新、园区建设、加大投入等关键举措。

必须更加注重改革开放。改革开放是引领经济发展的动力源泉，必须在加大改革力度、扩大双向开放上狠下功夫。坚决打好供给侧结构性改革攻坚战，完成“三去一降一补”重点任务。深化“放管服”改革，探索“多证合一”，实施“双随机一公开”，减少对企业生产经营干预，缩减行政审批事项，优化营商环境。深化国资国企市场化改革，加快推进市属经营性国有资产集中统一监管运营。深化财税体制改革，完善市以下财政体制。抓好县以下机关公务员职务与职级并行制度改革。

持续加大招商引资力度，围绕优势主导产业集群发展，发挥泰山国际登山节品牌效应，瞄准国内外500强、央企和知名民企，瞄准产业链前端、价值链高端，精准对接资本市场、投资基金和行业龙头，着力引进一批大资本、大基金和大企业、大集团。最大限度发挥好企业主体招商、县市区主阵地招商作用和市直部门招商职能优势，突出质量和效益，突出签约和落地，突出招商引资与招才引智并举，加大督查、考核、激励力度，搭建全市招商引资竞争大擂台，在全市营造聚心招商、用力招商浓厚氛围。

全力推动外贸稳增长调结构，注重发展外贸新业态新模式，引导企业加快发展跨境电商，推动离岸服务外包快速发展，培植外贸新的增长点。深度参与“一带一路”建设，加快企业“走出去”步伐，支持高端智能纺织装备、输变电设备、矿山装备等优势产能到境外投资办厂、开发资源、开拓市场，推动电力装备、能源、房建等优势企业，积极承接境外基础设施项目。加快泰安高新区海关综合监管区和青岛保税港区泰安（新泰）功能区建设。主动参与全省“一圈一带”发展战略，注重发挥好泰安与省会相近相融优势，做足做好 “发挥同城效应，借势借力发展”的文章。

必须更加注重科技创新。科技创新是一个企业、一个地区的竞争力和生命力所在，必须始终强化创新引领，深入实施创新驱动发展战略，加快创新型泰安建设步伐。高度重视抓好产学研合作机制创新，与国内外重点大学、重点科研院所、重点科技创新型企业开展合作，建设大的研发创新平台。近期拟重点建设泰安复合材料产业技术研究院、中国运载火箭高端技术应用研究院、石墨烯技术应用研究院、华大基因应用研究所等高端研发创新平台；依托泰安高新区国家高创中心，加快推进“泰山创新谷”建设。强化企业创新主体地位，围绕建设省级自主创新示范区，坚持以实施重大科技项目为支撑，鼓励企业加大研发投入，加强企业技术中心、工程实验室、工程技术研究中心、重点实验室和科技孵化器建设。加大对科技型中小企业支持力度，培育一批发展潜力大、成长性好、技术含量高的科技“小巨人”。高度重视创新人才队伍建设，尤其是充分发挥驻泰高校、科研单位作用，搭建高端人才创新创业交流平台和示范基地，培育和引进一批产业领军人才、科技人才、高技能人才。深化职称制度改革，健全对人才的股权、期权及分红激励机制。大力优化创新环境，弘扬创新精神，培育创新文化，让创新在全社会蔚然成风。

必须更加注重园区建设。我市各类园区普遍存在规模小、实力弱、功能不完善、体制不顺畅、特色不明显等问题，必须坚持园区建设与城市发展走向一致、与产业布局高度契合原则，突出创新、绿色、开放理念，高度重视抓好各级各类园区功能整合、建设提升和转型升级，谋划发展、培育形成一批有规模、有实力、有特色、有重大拉动作用的大园区、强园区。加快泰安高新区高科技产业园建设，努力拓展发展空间。新泰经济开发区、肥城高新技术产业开发区积极争创国家级园区。创新园区体制机制，重点创新园区干部队伍配备机制，打造一支基层经验丰富、法制意识强的群众工作队伍，一支懂经济、外向型、善沟通的招商引资队伍，一支事业心强、亲和力强、服务意识强的项目建设队伍。创新园区创业载体，实现由引资引项目为主到引技术引人才团队为主的转变。创新园区资本运营平台，实现由行政管理主导招商到市场经营、资本招商的转变。创新园区服务模式，提高园区服务效能和软实力。强化园区考核激励，将园区建设、转型升级、带动作用等纳入全市科学发展考核，推动园区发展实现新突破。

必须更加注重加大投入。投资仍然是当前和今后一个时期拉动经济发展的主要力量，必须始终把项目建设作为发展的“生命线”，下大气力抓好一批重大项目，不断增强经济发展后劲。重点推进投资100亿元的泰安抽水蓄能电站二期工程、投资200亿元的新泰采煤沉陷区光伏领跑技术基地等支撑撬动力强的重大产业项目，以及部分投资过50亿元、30亿元的重大财源项目建设。积极实施投资130亿元的新泰至梁山高速公路泰安段、投资60亿元的济南至泰安高速公路泰安段、投资48亿元的青兰高速泰安至东阿段、投资35亿元的京沪高速公路改扩建泰安段等拉动全局的重大基础设施工程。做好济泰城际铁路、聊泰铁路、中兴电力东平电厂、泰安机场、东平湖湖区航道、泰新高速公路扩容等项目前期准备工作。深度策划100个亿元以上重大项目，按照“三强”标准筛选确定120个左右市级重点建设项目、100个前期推进项目，采取有力措施，加快实施推进。

高度重视投融资平台建设，坚持市场化手段，创新“财政+基金+金融”投融资模式，密切与省财金集团合作，加快推进525亿元合作协议的落地，发挥好对泰安经济的牵引拉动作用。特别是谋划好、运作好150亿元的产业投资基金群，用好与兖矿集团合作设立的100亿元融资租赁基金，为我市实体经济发展和基础设施建设提供资金支持。加强融资担保平台建设，做大做强国泰民安、金融控股、泰山旅游、泰山科技四大国有资本投资运营平台，增强为企业担保、续贷、续保、增信支持的能力。

更大力度抓好金融服务，构建多层次、多样化、广覆盖的政银企合作体系，年内银企合作签约额力争突破300亿元。用好银税互动平台，以信授信，解决企业融资难问题。加快泰安金融商务区建设。发展壮大泰安银行、农商银行等地方金融机构。完善支持小额贷款公司、民间融资机构、融资性担保机构、金融控股集团等政策措施，深入推进新型农村合作金融改革试点。出台政策，加大力度，鼓励企业在境内外上市和在“新三板”挂牌融资。建立金融债务风险联合处置和信息通报机制，有序开展“破圈断链”工作，坚决守住不发生系统性区域性金融风险底线。加快社会信用体系建设，打造诚信泰安。

创新完善政府工作机制

本届政府的主要职责就是抓好市十一次党代会确立的工作任务落实，“落实”二字重于泰山。全体政府工作人员必须以创先争优为动力，以机制建设为保障，全面履行好政府各项职能，不断提高政府自身建设水平。重点健全“五大工作机制”：

一是健全快速高效的工作推进机制，加快建设服务政府。成立“三强”企业培植、重点项目建设工作推进领导小组，建立季度现场办公制度，采取市县联动、领导包保、现场调度、联席会议等形式，为企业和项目提供全方位、个性化服务。健全经常性联系服务群众、企业机制，根据群众、企业所思所盼，列出政务服务问题清单，制定具体方案，认真加以解决。深入实施“互联网+政务服务”行动，推进政务服务实体大厅和全市综合数据平台建设，大力推动网上办事、掌上办事，让数据多跑路，让群众少跑腿。大力提升12345民生服务平台服务能力，切实解决好关系群众切身利益的热点难点问题。

二是健全依法规范的运行机制，加快建设法治政府。严格按照法定权限和程序行使权力、履行职责。全面实施“七五”普法规划，深入推进依法治市工作。健全科学民主决策机制，完善依法行政制度体系。修订并落实好《市政府工作规则》，严格项目预审、土地利用、投融资管理等委员会议事规则。健全行政权力监督制约机制，主动接受市人大及其常委会的法律监督、工作监督和市政协的民主监督，加强与各民主党派、工商联和各人民团体、社会各界的沟通联系，高质量办理人大代表议案、建议和政协提案。完善行政执法体制，推动跨部门、跨行业综合执法。大力推进政务公开，完善政府新闻发布制度，及时主动回应社会关切，保障群众的知情权、参与权和监督权。

三是健全比学赶超的激励机制，加快建设责任政府。完善目标责任考核机制，对各项工作都列出专题、制定专案、明确专人，具体到项目上，落实到台账中，逐一压实责任，层层传导压力，搭建起“比学赶超”竞争擂台。改进重点项目观摩考核机制，重点考核财源项目、工业项目、旅游文化项目等经济类实体项目，引导各级把主要精力用到财源建设上。建立严明严厉督查调度制度，确保各项工作落实到位、富有成效。在重大项目建设、招商引资等急难险重任务中发现、识别、使用干部，建立正向激励和容错纠错机制，旗帜鲜明地为敢于担当的干部担当、为敢于负责的干部负责。

四是健全作风转变的引领机制，加快建设务实政府。牢牢抓住想干事、敢干事这两个关键点，大力倡树“不找借口、争创一流”的进取精神，对各项任务都必须用一流标准来要求、一流水平来衡量。强化时间观念和效率意识，凡事都必须雷厉风行，做到先人一步、快人一拍，以废寝忘食、事不过夜的态度推进工作落实。各级领导干部要以上率下、靠前指挥，敢于担责、勇于担险，对重点工作、疑难问题要盯上靠上，不回避、不推诿、不敷衍、不懈怠，敢于应对重大挑战、抵御重大风险、克服重大阻力、解决重大矛盾，以功成不必在我的责任担当，确保中央和省市委各项决策部署落地生根。

五是健全从严治政的监督机制，加快建设廉洁政府。牢固树立“四个意识”，严明政治纪律和政治规矩，带头遵守“三条例、两准则”和中央八项规定，驰而不息纠正“四风”。大力推进政府系统全面从严治党，严格落实主体责任，切实履行“一岗双责”。健全完善惩治和预防腐败体系，加强对重点领域、关键环节、重要岗位权力运行的监督制约。支持纪检监察机关监督执纪问责，支持审判检察机关独立行使职权，严肃查处违纪违法案件。政府每一位工作人员必须始终做到秉公用权、为民用权，崇廉尚实、清白干事，树立政府系统勤政清廉的良好形象。

各位代表，践行新理念、增创新优势、建设新泰安，关键在于敢想敢为。敢想才有机遇，敢为才能突破。让我们紧密团结在以习近平同志为核心的党中央周围，在中共泰安市委的坚强领导下，进一步加快工作节奏、加大工作力度、提升工作标准、提高工作效能，高扬干事风向标，撸起袖子加油干，为加快建设富裕文明幸福新泰安而努力奋斗！
